# Supplementary material for: Pectinases Secretion by Saccharomyces cerevisiae: Optimization in Solid-State Fermentation and Identification by a Shotgun Proteomics Approach
Source: Molecules. 2022 Aug 5;27(15):4981. doi: 10.3390/molecules27154981 (PMC9370124; doi:10.3390/molecules27154981)
Supplement: Supplementary file 1 [file molecules-27-04981-s001.zip › molecules-1758317-supplementary.pdf]

Supplementary Material

Pectinases Secretion by *Saccharomyces cerevisiae*: Optimization in Solid-State Fermentation and Identification by a Shotgun Proteomics Approach

Matheus Mikio Takeyama [1], Márcia Corrêa de Carvalho [1], Helena Sacco Carvalho [2], Cristiane Rodrigues Silva [1], Ana Paula Trovatti Uetanabaro [3], Andrea Miura da Costa [3], Joseph A. Medeiros Evaristo [4], Fábio César Sousa Nogueira [4,5], Ana Elizabeth Cavalcante Fai [1,6] and Maria Gabriela Bello Koblitz [1,*]

[1] Food and Nutrition Graduate Program (PPGAN), Federal University of the State of Rio de Janeiro (UNIRIO), Rio de Janeiro, RJ, Brazil; mikio_m@hotmail.com (M.M.T.); marcia.feagri@gmail.com (M.C.d.C.); cristiane.silva@unirio.br (C.R.S.); bethfai@yahoo.com.br (A.E.C.F.)
[2] Nutrition School, Federal University of the State of Rio de Janeiro (UNIRIO), Rio de Janeiro, RJ, Brazil; helenasacco@edu.unirio.br
[3] Department of Biological Sciences, Santa Cruz State University (UESC), Ilhéus, BA, Brazil; aptuetanabaro@gmail.com (A.P.T.U.); amcosta@uesc.br (A.M.d.C.)
[4] Laboratory of Proteomics/LADETEC, Institute of Chemistry, Federal University of Rio de Janeiro, RJ, Brazil; joseph.am.evaristo@gmail.com (J.A.M.E.); fabiocsn@iq.ufrj.br (F.C.S.N.)
[5] Proteomics Unit, Institute of Chemistry, Federal University of Rio de Janeiro, RJ, Brazil
[6] Department of Basic and Experimental Nutrition, Rio de Janeiro State University (UERJ), Rio de Janeiro, RJ, Brazil
* Correspondence: maria.koblitz@unirio.br; Tel./Fax: +55-21-2542-7236

Table S1. ANOVA for the influence of the tested variables on the secretion of total pectinolytic activity (U mL^{-1}), by *S. cerevisiae* strain, according to the factorial design.

	square sum	degrees of freedom	quadratic mean	F
regression	7.21	7	1.03	28.47
residue	0.43	12	0.04	
lack of fit	0.39	9		tabulated F
pure error	0.05	3		5% = 2.91
total	7.65	19		

$r^2 = 0.94$.

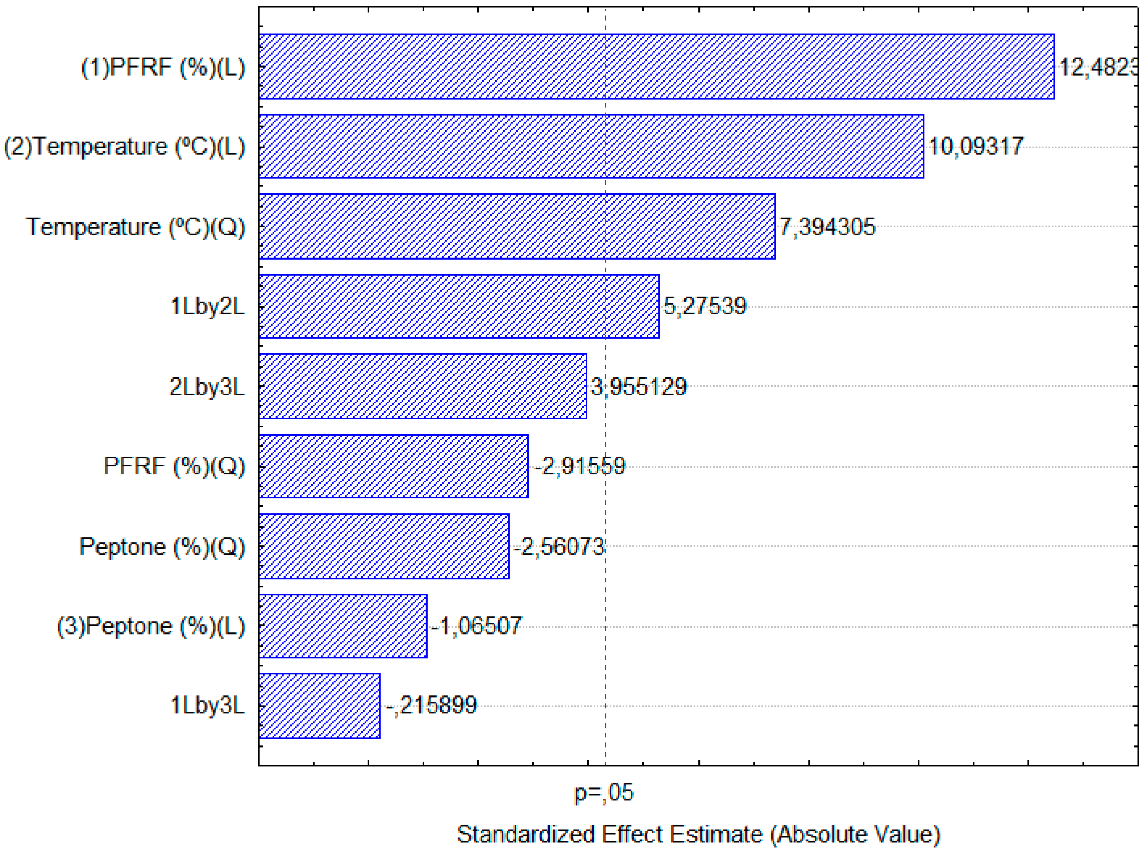

Figure S1. Pareto chart for the influence of the tested variables on the secretion of total pectinolytic activity (U mL^{-1}) by *S. cerevisiae* strain, according to CCRD - 1.

Table S2. ANOVA for the influence of the tested variables on the secretion of total pectinolytic activity (U mL^{-1}) by *S. cerevisiae* strain, according to CCRD - 1.

	**square sum**	**degrees of freedom**	**quadratic mean**	**F**
regression	36.38	4	9.09	5.73
residue	19.03	12	1.59	
lack of fit	18.83	10		tabulated F
pure error	0.20	2		5% = 3.26
total	55.41	16		

$r^2 = 0.71$

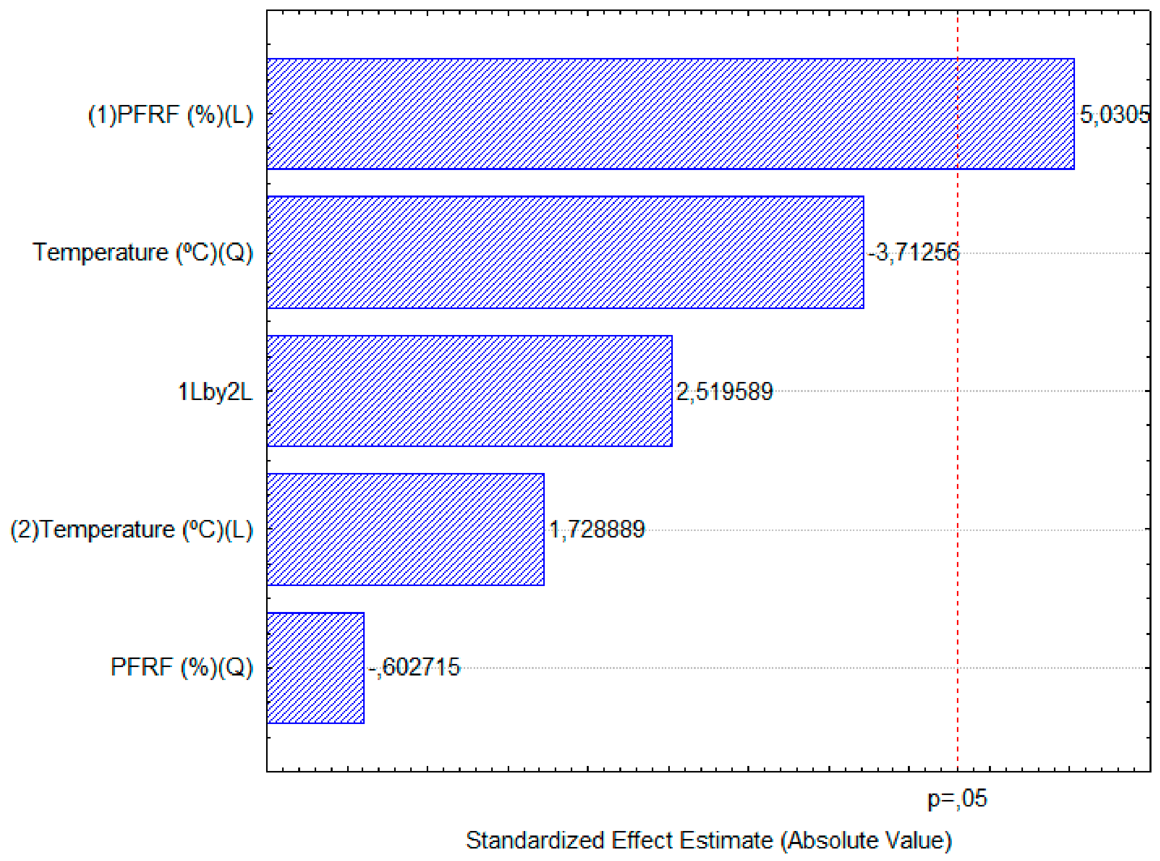

Figure S2. Pareto chart for the influence of the tested variables on the secretion of total pectinolytic activity (U mL^{-1}) by *S. cerevisiae* strain, according to CCRD - 2.

Table S3. ANOVA for the influence of the tested variables on the secretion of total pectinolytic activity (U mL^{-1}) by *S. cerevisiae* strain, according to CCRD - 2.

	square sum	degrees of freedom	quadratic mean	F
regression	38.14	5	7.63	6.80
residue	5.61	5	1.12	
lack of fit	4.04	3		tabulated F
pure error	1.57	2		5% = 5,05
total	43.75	10		

$r^2 = 0.87$

Table S4. Matrix for CCRD - 2, total pectinolytic activity response and expected results by the model descriptive equation, after 24 h of fermentation by *S. cerevisiae* strain.

Assay	PFRM (%)	Temperature (ºC)	U mL^{-1} (Observed)	U mL^{-1} (Expected)
1	12	15.5	0.86	0.97
2	12	32.5	0.80	0.00
3	52	15.5	0.86	1.90
4	52	32.5	5.26	5.22
5	4	24	0.31	0.94
6	60	24	6.06	5.36
7	32	12	0.86	0.07
8	32	36	0.86	1.60
9	32	24	4.42	3.59
10	32	24	3.70	3.59
11	32	24	2.66	3.59

Table S5. Matched proteins from *S. cerevisiae* database.

Accession	Description
P22943	12 kDa heat shock protein OS=Saccharomyces cerevisiae (strain ATCC 204508 / S288c) OX=559292 GN=HSP12 PE=1 SV=1
P00358	Glyceraldehyde-3-phosphate dehydrogenase 2 OS=Saccharomyces cerevisiae (strain ATCC 204508 / S288c) OX=559292 GN=TDH2 PE=1 SV=3
Q01855	40S ribosomal protein S15 OS=Saccharomyces cerevisiae (strain ATCC 204508 / S288c) OX=559292 GN=RPS15 PE=1 SV=1
P53334	Probable family 17 glucosidase SCW4 OS=Saccharomyces cerevisiae (strain ATCC 204508 / S288c) OX=559292 GN=SCW4 PE=1 SV=1
P46955	Beta-glucosidase-like protein NCA3, mitochondrial OS=Saccharomyces cerevisiae (strain ATCC 204508 / S288c) OX=559292 GN=NCA3 PE=3 SV=1
P38248	Cell wall protein ECM33 OS=Saccharomyces cerevisiae (strain ATCC 204508 / S288c) OX=559292 GN=ECM33 PE=1 SV=3
P02994	Elongation factor 1-alpha OS=Saccharomyces cerevisiae (strain ATCC 204508 / S288c) OX=559292 GN=TEF1 PE=1 SV=1
P04911	Histone H2A.1 OS=Saccharomyces cerevisiae (strain ATCC 204508 / S288c) OX=559292 GN=HTA1 PE=1 SV=2
P47001	Cell wall mannoprotein CIS3 OS=Saccharomyces cerevisiae (strain ATCC 204508 / S288c) OX=559292 GN=CIS3 PE=1 SV=1
P60010	Actin OS=Saccharomyces cerevisiae (strain ATCC 204508 / S288c) OX=559292 GN=ACT1 PE=1 SV=1
Q08969	Protein GRE1 OS=Saccharomyces cerevisiae (strain ATCC 204508 / S288c) OX=559292 GN=GRE1 PE=1 SV=1
Q12355	Cell wall mannoprotein PST1 OS=Saccharomyces cerevisiae (strain ATCC 204508 / S288c) OX=559292 GN=PST1 PE=1 SV=1
P05759	Ubiquitin-40S ribosomal protein S31 OS=Saccharomyces cerevisiae (strain ATCC 204508 / S288c) OX=559292 GN=RPS31 PE=1 SV=3
P36135	Probable secreted beta-glucosidase UTH1 OS=Saccharomyces cerevisiae (strain ATCC 204508 / S288c) OX=559292 GN=UTH1 PE=1 SV=2
P05737	60S ribosomal protein L7-A OS=Saccharomyces cerevisiae (strain ATCC 204508 / S288c) OX=559292 GN=RPL7A PE=1 SV=3
P0CX46	60S ribosomal protein L2-B OS=Saccharomyces cerevisiae (strain ATCC 204508 / S288c) OX=559292 GN=RPL2B PE=1 SV=1
P53301	Probable glycosidase CRH1 OS=Saccharomyces cerevisiae (strain ATCC 204508 / S288c) OX=559292 GN=CRH1 PE=1 SV=1
P22146	1,3-beta-glucanosyltransferase GAS1 OS=Saccharomyces cerevisiae (strain ATCC 204508 / S288c) OX=559292 GN=GAS1 PE=1 SV=2
P00044	Cytochrome c iso-1 OS=Saccharomyces cerevisiae (strain ATCC 204508 / S288c) OX=559292 GN=CYC1 PE=1 SV=2
P00445	Superoxide dismutase [Cu-Zn] OS=Saccharomyces cerevisiae (strain ATCC 204508 / S288c) OX=559292 GN=SOD1 PE=1 SV=2
P32340	Rotenone-insensitive NADH-ubiquinone oxidoreductase, mitochondrial OS=Saccharomyces cerevisiae (strain ATCC 204508 / S288c) OX=559292 GN=NDI1 PE=1 SV=1
P15703	Glucan 1,3-beta-glucosidase OS=Saccharomyces cerevisiae (strain ATCC 204508 / S288c) OX=559292 GN=BGL2 PE=1 SV=1
P07280	40S ribosomal protein S19-A OS=Saccharomyces cerevisiae (strain ATCC 204508 / S288c) OX=559292 GN=RPS19A PE=1 SV=2
P53629	Sterol O-acyltransferase 2 OS=Saccharomyces cerevisiae (strain ATCC 204508 / S288c) OX=559292 GN=ARE2 PE=1 SV=1

P00560	Phosphoglycerate kinase OS=Saccharomyces cerevisiae (strain ATCC 204508 / S288c) OX=559292 GN=PGK1 PE=1 SV=2
P05743	60S ribosomal protein L26-A OS=Saccharomyces cerevisiae (strain ATCC 204508 / S288c) OX=559292 GN=RPL26A PE=1 SV=3
Q12692	Histone H2A.Z OS=Saccharomyces cerevisiae (strain ATCC 204508 / S288c) OX=559292 GN=HTZ1 PE=1 SV=3
P04037	Cytochrome c oxidase subunit 4, mitochondrial OS=Saccharomyces cerevisiae (strain ATCC 204508 / S288c) OX=559292 GN=COX4 PE=1 SV=1
P0CX83	60S ribosomal protein L19-B OS=Saccharomyces cerevisiae (strain ATCC 204508 / S288c) OX=559292 GN=RPL19B PE=1 SV=1
P35997	40S ribosomal protein S27-A OS=Saccharomyces cerevisiae (strain ATCC 204508 / S288c) OX=559292 GN=RPS27A PE=1 SV=1
P05750	40S ribosomal protein S3 OS=Saccharomyces cerevisiae (strain ATCC 204508 / S288c) OX=559292 GN=RPS3 PE=1 SV=5
P40482	Protein transport protein SEC24 OS=Saccharomyces cerevisiae (strain ATCC 204508 / S288c) OX=559292 GN=SEC24 PE=1 SV=1
P28319	Cell wall protein CWP1 OS=Saccharomyces cerevisiae (strain ATCC 204508 / S288c) OX=559292 GN=CWP1 PE=1 SV=2
P40212	60S ribosomal protein L13-B OS=Saccharomyces cerevisiae (strain ATCC 204508 / S288c) OX=559292 GN=RPL13B PE=1 SV=1
P0CX26	60S ribosomal protein L43-B OS=Saccharomyces cerevisiae (strain ATCC 204508 / S288c) OX=559292 GN=RPL43B PE=1 SV=1
P0CX30	40S ribosomal protein S23-B OS=Saccharomyces cerevisiae (strain ATCC 204508 / S288c) OX=559292 GN=RPS23B PE=1 SV=1
P18239	ADP,ATP carrier protein 2 OS=Saccharomyces cerevisiae (strain ATCC 204508 / S288c) OX=559292 GN=PET9 PE=1 SV=2
P38616	Protein YGP1 OS=Saccharomyces cerevisiae (strain ATCC 204508 / S288c) OX=559292 GN=YGP1 PE=1 SV=2
P52489	Pyruvate kinase 2 OS=Saccharomyces cerevisiae (strain ATCC 204508 / S288c) OX=559292 GN=PYK2 PE=1 SV=1
P23776	Glucan 1,3-beta-glucosidase I/II OS=Saccharomyces cerevisiae (strain ATCC 204508 / S288c) OX=559292 GN=EXG1 PE=1 SV=1
P40472	Probable secreted beta-glucosidase SIM1 OS=Saccharomyces cerevisiae (strain ATCC 204508 / S288c) OX=559292 GN=SIM1 PE=1 SV=2
P32836	GTP-binding nuclear protein GSP2/CNR2 OS=Saccharomyces cerevisiae (strain ATCC 204508 / S288c) OX=559292 GN=GSP2 PE=1 SV=1
P40442	Putative uncharacterized protein YIL169C OS=Saccharomyces cerevisiae (strain ATCC 204508 / S288c) OX=559292 GN=YIL169C PE=3 SV=1
P00761 SWISS-PROT:P00761	Trypsin - Sus scrofa (Pig).
O76013	SWISS-PROT:O76013 Keratin, type I cuticular HA6 (Hair keratin, type I HA6)
P28496	Sphingosine N-acyltransferase LAC1 OS=Saccharomyces cerevisiae (strain ATCC 204508 / S288c) OX=559292 GN=LAC1 PE=1 SV=2
P0CX53	60S ribosomal protein L12-A OS=Saccharomyces cerevisiae (strain ATCC 204508 / S288c) OX=559292 GN=RPL12A PE=1 SV=1
P39998	Enhancer of mRNA-decapping protein 3 OS=Saccharomyces cerevisiae (strain ATCC 204508 / S288c) OX=559292 GN=EDC3 PE=1 SV=1

Table S6. Matched proteins from pectinase database.

Accession	Description
A0A0G4N991	Uncharacterized protein OS=Verticillium longisporum OX=100787 GN=BN1708_008936 PE=4 SV=1
A0A1Y2U259	Glycoside hydrolase family 55 protein OS=Hypoxylon sp. EC38 OX=1001937 GN=K449DRAFT_410365 PE=4 SV=1
A0A370JPG0	Uncharacterized protein OS=Venturia inaequalis OX=5025 GN=Vi05172_g1685 PE=3 SV=1
A0A395GPQ8	Pectin lyase-like protein (Fragment) OS=Aspergillus ibericus CBS 121593 OX=1448316 GN=BO80DRAFT_321211 PE=4 SV=1
A0A0L1JA42	Pectin lyase F OS=Aspergillus nomius NRRL 13137 OX=1509407 GN=ANOM_002499 PE=3 SV=1
A0A1V6V1X2	Uncharacterized protein OS=Penicillium coprophilum OX=36646 GN=PENCOP_c002G06905 PE=4 SV=1
A0A1Y2DZG1	Uncharacterized protein OS=Neocallimastix californiae OX=1754190 GN=LY90DRAFT_668057 PE=4 SV=1
A0A367IZ94	Uncharacterized protein (Fragment) OS=Rhizopus stolonifer OX=4846 GN=CU098_001619 PE=3 SV=1
X8JKF7	Pectate lyase OS=Rhizoctonia solani AG-3 Rhs1AP OX=1086054 GN=RSOL_437720 PE=4 SV=1
A0A2J6QBA4	Glycoside hydrolase family 55 protein OS=Pezoloma ericae OX=1745343 GN=NA56DRAFT_643654 PE=4 SV=1
A0A167AVW5	Pectate lyase OS=Colletotrichum incanum OX=1573173 GN=CI238_10558 PE=4 SV=1
